# An electrolyte flip flop - a patient with chronic hyperkalemic acidosis presenting with severe hypokalemic alkalosis

**DOI:** 10.1016/j.heliyon.2022.e12607

**Published:** 2022-12-24

**Authors:** Avital Angel-Korman, Rey Biton, Vladimir Rappoprt, Michael Hausmann, Adi Leiba

**Affiliations:** a Faculty of Health Sciences, Ben Gurion University, Beer Sheva, Israel; b Institute of Nephrology and Hypertension, Samson Assuta Ashdod University Hospital, Ashdod, Israel

**Keywords:** Alkalosis, Sodium polystyrene sulphonate, Hypokalemia, Hypernatremia, Electrolytes

## Abstract

A 76-year-old man was evaluated in our emergency department (ED) for right toe swelling and pain. His initial ED workup revealed volume overload, uncontrolled hypertension, slow atrial fibrillation, refractory hypokalemia, mixed metabolic alkalosis and respiratory acidosis, with a normal plasma pH, and hypernatremia.

His medical chart revealed long standing hyperkalemia and metabolic acidosis, related to his diabetic kidney disease. We hypothesized that a short course of daily SPS ingestion (Sodium Polystyrene Sulfonate, “Kayexalate”) was the sole etiology for the compound electrolyte abnormalities and the electrolyte “flip flop”.

SPS ingestion can cause hypokalemia by excessive potassium binding in the gut. SPS exchanging potassium for sodium caused excessive sodium retention leading to hypernatremia, hypertension and volume overload. Volume overload worsened his chronic obstructive sleep apnea and yielded respiratory acidosis.

Finally hypokalemia by itself was the main trigger for generation and maintenance of metabolic alkalosis. Urinary electrolytes, and renin and aldosterone levels taken at the ED ruled out primary aldosteronism and renal potassium and hydrogen loss.

The patient's potassium was replenished by both PO and IV routes. He was treated for his volume overload and hypertension with furosemide. Spironolactone and amiloride, potassium sparing diuretics, were cautiously given only during his hypokalemic phase. His plasma sodium and potassium levels, blood pressure and volume status gradually improved.

“Kayexalate” effect should be suspected in a patient presenting with unexplained hypokalemia and alkalosis, accompanied by volume overload rather than volume depletion, developing shortly after SPS ingestion. ED doctors should specifically ask CKD or ESRD patients on SPS, as it otherwise can skip the medication reconciliation process.

## 1 Introduction

A 76-year-old man was sent to our emergency department (ED) with the chief complaint of right toe swelling and pain. He denied fever, vomiting or diarrhea.

His past medical history is evident for chronic kidney disease (CKD), benign prostatic hypertrophy (BPH), hypertension, hyperlipidemia, diabetes mellitus, morbid obesity, obesity hypoventilation syndrome (OHS), peripheral vascular disease (PVD), s/p left below knee amputation (BKA), ischemic heart disease (IHD) with previous coronary revascularization, history of atrial fibrillation and remote heavy smoking.

His home meds included lercanidipine, bisoprolol, rosuvastatin, apixaban, pregabalin, solifenacin, esomeprazole, and sertraline. He was on two alpha blockers-doxazosin and tamsulosin in a combination pill with dutasteride. He was not taking thiazides, loop diuretics or any other diuretic medication.

On arrival to the ED, he was alert and oriented, in no acute distress and afebrile. His blood pressure upon arrival was 177/52 mmHg. He had slow atrial fibrillation with a few short pauses.

His exam revealed central obesity, reduced breath sounds bilaterally, remote heart sounds and pitting edema +2 of his right ankle and knee as well as redness, tenderness and swelling of his right toe.

In the ED the patient underwent bedside point of care US (POCUS) which revealed preserved systolic and diastolic cardiac function and non compressible inferior vena cava, suggesting volume overload.

Pertinent laboratory findings were severe hypokalemia (2.3 mmol/L), acute on chronic renal failure (creatinine- 2.3 mg/dL), metabolic alkalosis (blood gas bicarbonate- 43.5 mmol/L) , respiratory acidosis (pCO2-80.7mmHg) with normal blood pH (7.35) and hypernatremia of 150 mmol/L. Glucose levels were 138 mg/dL. Magnesium- 2 mg/dL, calcium - 8.26 mg/dL.

Urine studies revealed urine sodium of 50 mmol/L, urine chloride of 48.7 mmol/L, low urinary potassium (13 mmol/L), and a positive urinary anion gap (Table 1). His urine was acidic (urine pH of 5.5) despite metabolic alkalosis. Urine potassium to creatinine ratio was 21.5 mmol/g.

**Table 1:** Results of laboratory tests prior to admission (as an outpatient), on arrival to the ED and after 1 month.

	3 weeks prior to admission (outpatient)	ED visit	One month later
Creatinine (mg/dL)	1.59	2.3	4.5
Urea (mg/dL)	93.7	87.4	183.4
Sodium (mmol/L)	142.5	150	132
Potassium (mmol/L)	6.6	2.3	4.4
Chloride (mmol/L)	N/A	106	99
pH	7.18	7.35	7.35
Bicarbonate(mmol/L)	18.7	43.5	24.9
pCO2 (mmHg)	50.7–54	80.7	46.4
Anion gap (mmol/L)	N/A	3 (eAG-7)	8 (eAG- 7)
Urinary chloride (mmol/L)	N/A	48.7	N/A
Urinary Sodium (mmol/L)	N/A	50	N/A
Urinary potassium (mmol/L)	N/A	17	N/A
Urinary anion gap (mmol/L)	N/A	18.3	N/A

Aldosterone levels were measured in a supine position in the ED and were low (3.24 ng/dl). Cortisol levels were within normal limits. Direct renin level was not suppressed at 11.06 mcIU/ml.

The nephrology consultant and ED staff were looking at his medical records, that surprisingly revealed a recent (3 weeks prior to ED visit) creatinine of 1.6 mg/dL, hyperkalemia (6.6 mmol/L), normo-natremia (Na- 142.5 mmol/L) and a combined metabolic and respiratory acidosis (pH- 7.18, Bicarbonate 18.7 mmol/L, pCO2 50.7–54 mmHg) (Table 1). He had chronic tendency for hyperkalemia and normal anion gap acidosis tentatively diagnosed as type IV RTA (related to diabetic hyporeninemic hypoaldosteronism). We realized the patient was started on “Kayexalate” 15 g BID 2 weeks prior to his ED visit due to hyperkalemia, for which he was fully adherent.

During his hospital stay he had persistent metabolic alkalosis (bicarbonate up to 42.3 mmol/L). Only after his hypokalemia and respiratory acidosis resolved (K – 4.4 mmol/L, pCO2 – 46.4 mmHg - Table 1), after a month of hospital stay, his bicarbonate decreased to 24.9milimol/liter (Table 1).

## 2 Discussion

The combination of hypertension, hypokalemia and metabolic alkalosis led us to an initial “working diagnosis” of primary aldosteronism (PA) [1, 2, 3, 4]. Nevertheless, PA was ruled out due to low aldosterone levels and non-suppressed renin levels.

As we later realized that his electrolyte derangements appeared in the last three weeks, moving from a state of hyperkalemia-metabolic acidosis to hypokalemia-metabolic alkalosis, along with worsening of baseline respiratory acidosis and appearance of hypernatremia, we suspected the newly prescribed sodium polystyrene sulfonate (“Kayexalate”- SPS) to be the etiology for his “flip-flop” electrolyte abnormalities. Kayexalate (SPS), combined with low oral intake, can cause hypokalemia by excessive potassium binding in the gut [5]. Moreover, we assume that SPS exchanging potassium for sodium likely caused excessive sodium retention leading to hypernatremia, hypertension and volume overload [6].

No readily identifiable cause of metabolic alkalosis was evident (no vomiting, volume contraction or use of diuretics). The patient was never intubated, his pCO2 gradually decreased and no chloride depletion was noted, so post hypercapnic alkalosis can be excluded. There was no ingestion of bicarbonate or bicarbonate “potential”, i.e. citrate. Finally, there was no gastric or excessive renal loss of protons or potassium, as his aldosterone was low, as well as his urinary potassium [7].

Despite alkalosis, excess bicarbonate was proximally reabsorbed (yielding paradoxical aciduria i.e. a low urine pH of 5.5).

We hypothesized that hypokalemia by itself was the trigger both for worsening renal function as well as for bicarbonate generation [8]. Hypokalemia causes a cellular shift, with protons entering the cells in exchange for potassium leaking out of the cells [9]. Alkalosis is further aggravated when such a shift occurs in the renal proximal tubular cells, causing intracellular acidification, increased renal ammonium production, renal citrate absorption and proximal and distal tubular acidification [10].

Both hypokalemia and respiratory acidosis augment bicarbonate reclamation despite metabolic alkalosis inhibit the ability of bicarbonate reclamation despite metabolic alkalosis, creating a vicious cycle of alkalosis maintenance [10].

SPS overdose should be suspected in a patient presenting with unexplained hypokalemia and alkalosis, accompanied by volume overload.

## Declarations

### Author contribution statement

All authors listed have significantly contributed to the investigation, development and writing of this article.

### Funding statement

This research did not receive any specific grant from funding agencies in the public, commercial, or not-for-profit sectors.

### Data availability statement

Data included in article/supp. material/referenced in article.

### Declaration of interest’s statement

The authors declare no competing interests.

### Additional information

No additional information is available for this paper.
